# Femtosecond Double-Pulse Laser Ablation and Deposition of Co-Doped ZnS Thin Films

**DOI:** 10.3390/nano10112229

**Published:** 2020-11-10

**Authors:** Ignacio Lopez-Quintas, Esther Rebollar, David Ávila-Brande, Jesús G. Izquierdo, Luis Bañares, Carlos Díaz-Guerra, Ana Urbieta, Marta Castillejo, Rebeca de Nalda, Margarita Martín

**Affiliations:** 1 Instituto de Química Física “Rocasolano”, Agencia Estatal CSIC, Serrano 119, 28006 Madrid, Spain; e.rebollar@csic.es (E.R.); marta.castillejo@iqfr.csic.es (M.C.); mmm@iqfr.csic.es (M.M.); 2 Departamento de Química Inorgánica, Universidad Complutense de Madrid, 28040 Madrid, Spain; davilabr@ucm.es; 3 Departamento de Química Física, Universidad Complutense de Madrid, 28040 Madrid, Spain; jegonzalez@ucm.es (J.G.I.); lbanares@ucm.es (L.B.); 4 Departamento de Física de Materiales, Facultad de Ciencias Físicas, Universidad Complutense de Madrid, Ciudad Universitaria s/n, 28040 Madrid, Spain; cdiazgue@ucm.es (C.D.-G.); anaur@ucm.es (A.U.)

**Keywords:** ultrashort laser pulses, double pulse irradiation, pulsed laser deposition, thin films, nanoparticles, diluted magnetic semiconductors, II-VI semiconductors, transition metal doping, cobalt, zinc sulfide

## Abstract

Nanostructured thin films of Co-doped zinc sulfide were synthesized through femtosecond pulsed laser deposition. The scheme involved ablation of physically mixed Co and ZnS with pairs of ultrashort pulses separated in time in the 0–300 ps range. In situ monitorization of the deposition process was carried out through a simultaneous reflectivity measurement. The crystallinity of generated nanoparticles and the inclusion of Co in the ZnS lattice is demonstrated by transmission electron microscopy and energy dispersive X-ray microanalysis (TEM-EDX) characterization. Surface morphology, Raman response, and photoluminescence of the films have also been assessed. The role of interpulse temporal separation is most visible in the thickness of the films obtained at the same total fluence, with much thicker films deposited with short delays than with individual uncoupled pulses. The proportion of Co in the synthesized doped ZnS nanoparticles is found to be substantially lower than the original proportion, and practically independent on interpulse delay.

## 1. Introduction

From the late 1970s a family of materials known as diluted magnetic semiconductors (DMS) have received great interest because of their potential in technological applications like optoelectronics and data storage [1]. In DMS, a small fraction of the atoms forming the lattice of a semiconductor is replaced by transition metal atoms such as Fe, Mn, Co, or Ni. The idea behind DMSs is that the low percentage of doping magnetic atoms allows to maintain the desirable electronic and optical semiconductor properties while at the same time incorporating a magnetic response. This characteristic of DMS has fostered the study of these materials as candidates for the fabrication of spintronic devices [2,3,4]. The huge research effort has been directed mainly to increasing the Curie temperature of the resulting material, T_c_, to above room temperature, which is a prerequisite for practical applications [5]. The first steps were taken by Ohno et al., who in 1996 reported a Curie temperature of 110 K for (Ga, Mn) As [6], and for this same material Tc was raised to 200 K through nanoengineering [7]. The prediction of Deitl et al. that reaching room temperature was feasible [8,9] and the successful experiment by Matsumoto et al. [10] encouraged intense research activity, and by now room temperature ferromagnetism has been found in many semiconductors doped with transition or rare earth metals [11]. Interestingly, ferromagnetism has been reported in nanostructures of metal oxide semiconductors even in the absence of a magnetic dopant [12,13,14]. The mechanisms underlying room temperature ferromagnetism in diluted magnetic semiconductors is still the subject of debate.

The group of the II-VI semiconductors is one of the families studied as candidates for DMS behavior. Among them, zinc sulfide (ZnS) is of particular interest partly to a large bandgap (3.68 eV for the zincblende structure, 3.91 eV for the wurtzite structure) [15], which renders it particularly suitable for optoelectronic applications. In nanostructured forms, ZnS presents appealing properties, and a broad variety of morphologies (nanoparticles, nanotubes, nanowires) have been synthesized [16]. Among the dopants proposed for ZnS (Mn, Co, Ni), Co is particularly favorable because the ionic radius of Co^2+^ (0.72 Å) is very close to that of Zn^2+^, (0.74 Å), which facilitates the inclusion of the transition metal ions in the semiconductor lattice with low strain [17,18] so that Co can be present as a dopant of ZnS to high atomic percentages (up to 30 at%) without destroying the host lattice [19]. The versatility in relation to nanostructure, together with the possibility of transition metal doping, makes nanostructured Co:ZnS a particularly attractive material [20,21]. Even though the body of work currently available is not very extensive, room temperature ferromagnetism has been found in Co-doped ZnS nanowires [18], nanoparticles [22], and thin films [23].

Among the procedures applied for the synthesis of DMS, pulsed laser deposition (PLD) is one of the preferred techniques to deposit high-quality thin films [24,25,26,27]. Based on laser ablation, PLD allows the often-stoichiometric transfer of multielemental targets to chosen substrates, it allows a fine control on the characteristics of the deposited film and deposition can take place at much lower temperatures than other film growth methods.

PLD can be particularly suitable for the synthesis of thin films of DMS since the nonequilibrium nature of the interaction can help increase the dopant concentration beyond the solubility limit at thermal equilibrium [28]. Control of the dopant proportion in the semiconductor lattice or the characteristics of the nanostructure of the deposits would open the possibility of modifying the material properties in terms of their optical and magnetic response [29,30,31]. Examples of successfully synthesized DMS-type materials through PLD include Co:ZnO [32,33,34], Mn:ZnO [28,35,36], Mn:SnO_2_ [37], and Ni:ZnO [38]. The preparation methods in these works differ, from ceramic techniques through physically mixed powders to separate targets of the host and the dopant that are ablated simultaneously and their plasmas are allowed to co-expand.

Scarce work exists on PLD of Co:ZnS [23,24,39]. Patel et al. [23,39] observed magnetic response in thin films of Co-doped ZnS grown with nanosecond UV pulses. They examined the morphology of the deposits in detail, concluding that texture (grain size, grain boundaries, and even the columnar growth observed for deposition at higher temperatures) played an important role on the reported magnetic properties. In [24], the authors employed nanosecond near-infrared laser pulses for the PLD process, reporting inclusion of Co atoms into both zincblende and wurtzite ZnS nanoparticles but a very weak magnetic response.

Laser ablation with ultrashort laser pulses, typically in the femtosecond range, allows the study of ultrafast phenomena in the target material using pump-probe experimental schemes [40,41], since the energy transfer from the electron to the lattice occurs in typically longer time scales than the pulse duration and thus the lattice remains unaffected during the pulse interaction [42,43]. Additionally, this non-equilibrium regime favors the formation of ablation products, like aggregates and nanoparticles, which cannot be easily obtained with longer pulses [44,45].

Taking advantage of the characteristics of ultrashort laser pulses, complex irradiation schemes, including tailored pulse shapes and pulse sequences, have been explored in order to modify the laser-matter interaction processes involved [46,47], with applications in fields such as the chemical analysis of materials [48,49,50], materials processing [51,52], and materials synthesis through PLD [53,54,55], among others. Pulse shaping techniques have been implemented in PLD experiments with the aim of controlling the characteristics of the deposits through the control of the ablation process and plasma expansion. In a similar way, ultrashort double pulse (DP) irradiation schemes have been also implemented in PLD but, to the best of our knowledge, only to a limited extent [56,57].

In a previous work [58] we investigated the double pulse femtosecond ablation dynamics of targets containing a mixture of metallic cobalt and zinc sulfide (2% of Co in weight). By analyzing the composition using time-of-flight mass spectrometry (TOF-MS) of plasmas obtained by DP irradiation at different interpulse delays (from 0 to 300 ps), a different ablation dynamics for the ionic species present in the plasma, including S^+^, Co^+^ and Zn^+^ as well as clusters containing these elements, were observed. A similar approach has been recently used for the study of DP ablation of magnesium targets, where plasma composition was analyzed as a function of the interpulse delay [49]. The observations suggest the possibility of exerting a certain degree of control on the plasma composition by controlling the delay between pulses, which would in turn cause changes in the composition and nanostructure of thin films prepared by fs-PLD.

In the present work, the aforementioned DP femtosecond irradiation approach was transferred to a PLD experiment with two objectives in mind; first, to obtain thin films containing crystalline ZnS nanoparticles doped with cobalt atoms, starting from the two separated components physically mixed together into a single target, and compare with results obtained in nanosecond PLD experiments; second, to evaluate the role of delay in the double-pulse femtosecond irradiation scheme on the composition, thickness, and nanostructure of the PLD-grown thin films.

## 2. Materials and Methods

**Figure 1:**
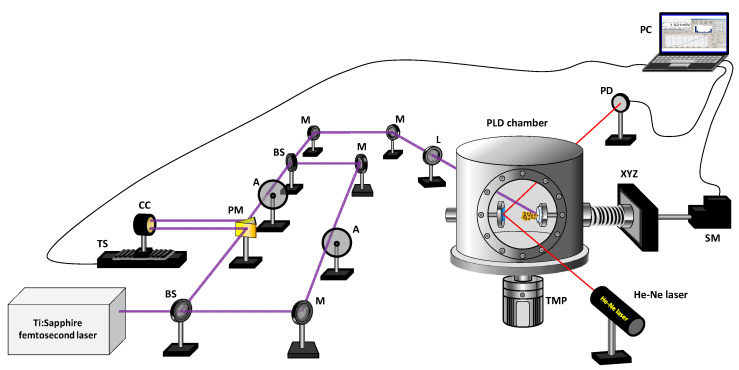
Scheme of the experimental set-up for double pulse femtosecond pulsed laser deposition (PLD). Symbols in the figure correspond to: TS = translation stage, CC = corner cube, BS = beam splitter, PM = prism mirror, M = mirror, A = attenuator, L = focusing lens, TMP = turbomolecular pump, PD = photodiode, XYZ = xyz positioning system, SM = step motor, PC = computer.

A target material composed by a mixture containing Co (10% in weight) and ZnS powders was used. Commercial ZnS (99.99% purity) and metallic Co (99.998% purity) powders were mechanically mixed and pressed in form of pellets. Irradiation of the targets was performed at an incidence angle of 55° with a Chirped Pulse Amplified Ti:Sapphire laser system (800 nm, 1 mJ per pulse, 100 fs, 1 kHz repetition rate). A scheme of the experimental set-up can be found in Figure 1. The linearly polarized laser beam was focused with an *f* = 30 cm focal length lens, with a beam waist at the focus (radius at 1/e^2^) of 30 ± 2 μm.

Ablation was performed by sequences of two individual pulses temporally separated by a controllable delay. This configuration will be named DP, for double-pulse irradiation. In DP conditions, the output of the laser system was split into two individual pulses with a modified Mach-Zehnder interferometer. This arrangement allows accurate control of the interpulse delay in the range of 0 to 300 ps. The spatial overlap of the beams coming out from the interferometer was ensured before each PLD experiment. The same applies for the temporal overlap; in this case a region of about 200 fs, in which the characteristic interference pattern between the temporally overlapped beams is observed, was considered as the delay zero and served as a reference before the growth of each deposit. Results in DP conditions were compared with those obtained with sequences of single pulses generated by blocking one of the arms in the interferometer, a configuration that will be named SP (single-pulse irradiation). The number of individual laser pulses (in SP irradiation) or double pulse sequences (in DP irradiation) was varied from 0.5 × 10^6^ to 2 × 10^6^. In order to avoid excessive cratering, the targets were rotated and displaced laterally during the deposition process with the help of a motorized *xyz* positioning system controlled by a dedicated software. The energy of each individual pulse was controlled with variable attenuators. In the experiments, the fluence of each individual pulse was set to ~0.2 J cm^−2^ (both for SP and DP scenarios). These conditions were found to be close to the ablation threshold for single pulse irradiation. This point will be discussed in more detail in the Results section. Ablation and deposition were performed under vacuum conditions (10^−5^ mbar) at room temperature. As substrates, pieces of Si (100) wafers and Transmission Electron Microscopy (TEM) grids, placed at 4 cm in front of the target, were used. The deposition process was monitored in situ by following the reflectance changes ongoing on the silicon substrates as the deposited layer was grown. For that purpose, a CW He-Ne laser (λ = 632.8 nm) was directed towards the substrate surface at an angle of ≈80° and the averaged power of the reflected beam was registered with a photodiode at 1 s intervals. These measurements helped us to determine the desired under-threshold conditions for each individual pulse. More details on this method are provided in the Results section. The thickness of the obtained deposits was measured by Atomic Force Microscopy (AFM). In order to perform these measurements, a small area of the Si substrates was masked prior to the deposition process and the height of the sharp step left after removing the mask was measured with a Bruker AFM Multimode 8 microscope equipped with a Nanoscope V controller in the tapping mode. The same equipment was employed to measure the roughness of the deposits at the nanometer scale. The morphology and composition of the deposited material was further studied by Transmission Electron Microscopy (TEM) coupled to X-ray Energy Dispersive Spectroscopy (EDX) with a JEOL JEM 2100 microscope coupled to an Oxford Inca analyzer operating at 200 kV with a point resolution of 0.25 nm. Micro-Raman and photoluminescence measurements were carried out at room temperature in a Horiba Jovin-Ybon LabRAM HR800 system. The samples were excited by a 325 nm He-Cd laser on an Olympus BX 41 confocal microscope. A charge coupled device detector was used to collect the scattered light. The spectral resolution of the system used was ~1.5 cm^−1^.

## 3. Results and Discussion

**Figure 2:**
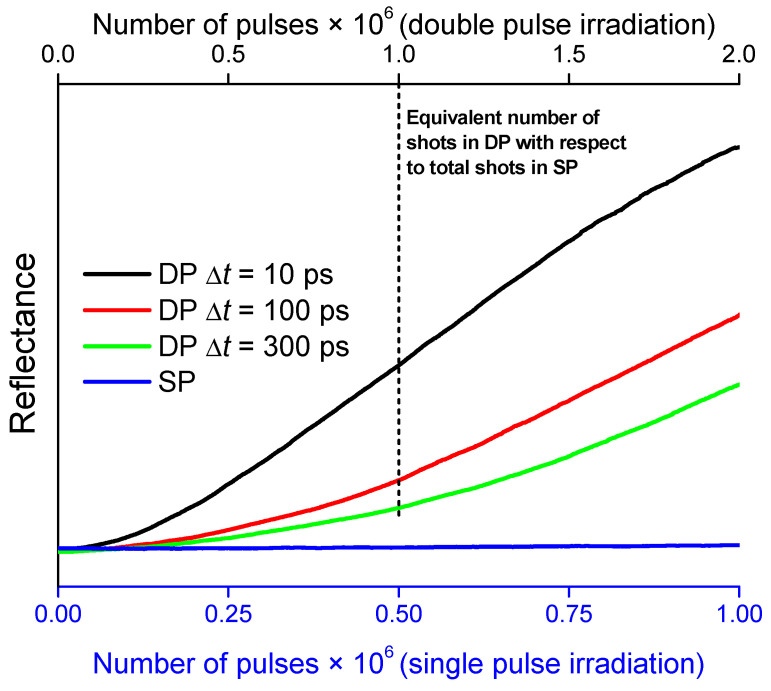
Evolution of the reflectance of a CW He-Ne laser beam as a function of the number of laser pulses for samples prepared by single pulse (SP) and double pulse (DP) irradiation at 10, 100, and 300 ps of interpulse delay. The vertical dashed line indicates the point where an equivalent number of individual laser pulses is achieved in the case of DP irradiation with respect to the total number of pulses in the SP case (1 × 10^6^ individual pulses). Note that the total number of pulses is 2 × 10^6^ for DP irradiation and 10^6^ for SP irradiation.

**Figure 3:**
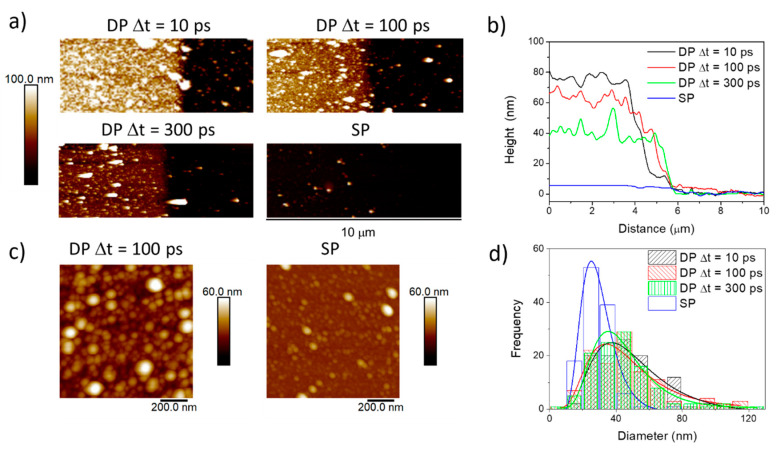
(**a**) Atomic Force Microscopy (AFM) images obtained in deposits prepared by DP and SP irradiation with 1 × 10^6^ double pulse sequences and 1 × 10^6^ individual pulses, respectively. (**b**) Example of AFM profiles obtained from the deposits. The profiles shown correspond to an average of around 10–20 individual profiles. (**c**) AFM topography images. (**d**) Size histograms of the nanoparticles and corresponding lognormal fitting (colour lines).

The pulsed laser deposition process was followed in situ by measuring the evolution of the deposited layer reflectance. This diagnostic helped us to establish irradiation conditions at the ablation threshold for individual pulses. In both SP and DP irradiation conditions, the fluence of each individual pulse was adjusted to a value that did not produce either a significant change in the reflectance measured on the silicon substrates during the deposition (Figure 2) or a significant deposit thickness measured ex situ by AFM (see Figure 3). This fluence value, of about 0.2 J cm^−2^, was in agreement with that found in our previous work as the ion detection threshold in TOF-MS measurements [58].

At this point, it should be noted that, for materials that are transparent at the incident radiation wavelength (632.8 nm) and at a fixed incidence angle, the intensity of the reflected beam can be calculated from the Fresnel coefficients. In this case, the variation of the reflectance R with the deposit thickness d results in a modulated signal [59] due to interference effects between the beam reflected on the first interlayer and the beam reflected on the second interlayer. The distance between the maxima and minima can be correlated with the thickness of the deposited layer if the refractive index is known. Therefore, the value of the reflectance at a given duration of the deposition process (equivalently, after a number of pulses) can provide an in situ estimation of the deposit thickness.

Figure 2 shows the evolution of the reflectance as a function of the number of laser pulses, measured on deposits prepared by SP and DP irradiation at 10, 100, and 300 ps of interpulse delay, from the start to the end of the PLD process, which is stopped at 10^6^ pulses for the SP case and 10^6^ sequences of two pulses for DP.

While the reflectance upon SP irradiation remains roughly constant after 10^6^ pulses (blue curve), it increases markedly in the DP case. This is a clear indication that SP conditions are below or around threshold for substantial material deposition, while DP conditions are significantly above the threshold, despite having the same single pulse fluence. Additionally, in the case of DP irradiation, an effect of the interpulse delay on the thickness of the deposits is observed. The thickest deposit was obtained by irradiation at the shortest interpulse delay of 10 ps (black curve in Figure 2); from that point, the thickness of the deposited layer decreases with the delay between pulses (red and green curves corresponding to 100 and 300 ps, respectively).

Due to the limited number of laser pulses applied, the first maximum expected in the reflectivity curves for the situation of constructive interference is not reached. However, additional deposits were prepared with a larger number of laser pulses. For those, the modulation of the reflectance signal did show the full modulation, which was compared with the expected curves. This is displayed in Figure S1 (see Supplementary Materials). Deposits were assumed to be transparent at the He-Ne laser wavelength, and therefore the refractive index of the deposited material was assumed to be equal to that of the pure ZnS, despite the confirmed presence of cobalt in the deposited material. The good fit of the experimental and theoretical curves at low deposit thickness (before first maximum) suggests that this assumption is a valid approach for correlating the changes in reflectance with thickness. Nevertheless, some deviation, which may be related with the presence of cobalt, is observed for higher thickness.

The effect of the DP vs. SP irradiation and the influence of the interpulse delay on the ablation yield were also observed through the ex situ analysis of the deposit thickness measured by AFM. Figure 3a shows the AFM images and Figure 3b shows the corresponding profiles of samples prepared by DP irradiation at interpulse delays of 10, 100, and 300 ps and by SP irradiation. Table 1 summarizes the average values found for the thickness of the thin films. Comparing the obtained thickness values for SP and DP irradiation, the coupling effect of the second pulse on the material is evidenced by the higher thickness achieved in the case of deposits obtained by DP irradiation.

The fact that shorter interpulse delays yield thicker deposits can be explained on the basis of the ablation mechanism of the main component ZnS, dominated by three photon absorption followed by energy and carrier depletion, as was described in [58]. The behaviour of film thickness as a function of delay is in agreement with the behaviour found in our previous paper, where shorter interpulse delays, in the region of 10–300 ps, produced a higher signal intensity in TOF-MS analysis of the plumes for all the ions studied. In relation with this effective decrease in fluence threshold in double pulse configuration, it is interesting to compare the behaviour observed here with that reported in [47] for Al, where the authors reported an increase of fluence threshold when two near IR pulses delayed in the time range 20–200 ps were employed for ablation. We believe that the fluence regime (around the single pulse threshold for our case, significantly above the threshold in Kudryashov’s work) plays an important role on this different behaviour, since the redeposition mechanism invoked in their case is hardly possible in the current situation.

The surface morphology and nanostructure of the deposits was also examined by AFM. Figure 3c shows the AFM topography images of the deposits obtained by SP and DP irradiation with interpulse delay of 100 ps. The size distribution of the nanoparticles, extracted from the AFM images, displayed in Figure 3d, were fitted with log-normal distributions obtaining average diameters of 47 ± 5, 43 ± 5 and 41 ± 2 nm for DP irradiation at 10, 100, and 300 ps, respectively, and 28 ± 1 nm for SP irradiation. Additionally, a small fraction of larger aggregates up to 120 nm is observed for DP irradiation at 100 ps delay.

**Figure 4:**
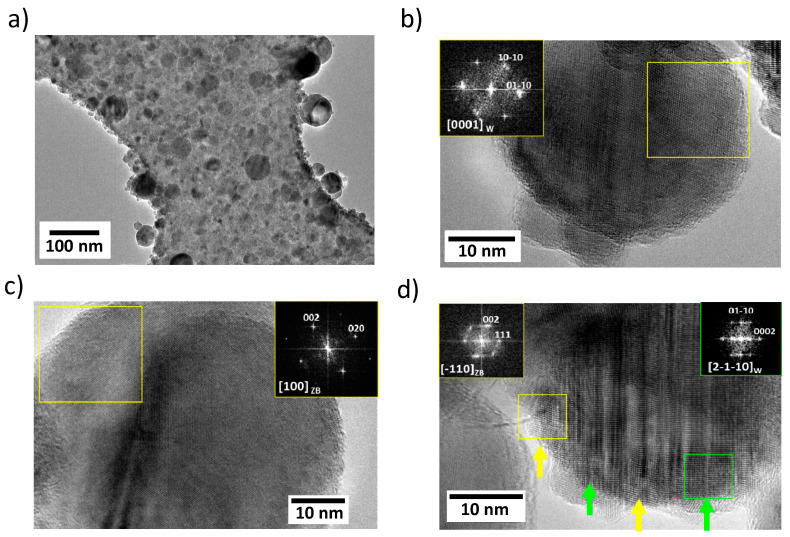
Transmission Electron Microscopy (TEM) images of the nanoparticles synthesized on polymer-coated TEM grids found in different PLD deposits. (**a**) shows an overview of one of the deposits where a large dispersion in the particle size can be observed. The deposit was obtained by DP irradiation at an interpulse delay of 10 ps and 2 × 106 double pulse sequences. In (**b**–**d**) high magnification images allows the identification of the crystalline phases in single nanoparticles obtained by DP irradiation at an interpulse delay of 300 ps and 2 × 106 double pulse sequences. (**b**–**d**) show TEM images together with the corresponding FFT patterns obtained (insets) in the areas highlighted with the coloured squares. In (**b**) the nanoparticle is identified as wurtzite along the [0001]_W_ zone axis. In (**c**) the nanoparticle is oriented along the [100]_ZB_ zone axis of cubic zinc blende. The several crystalline planes observed correspond to the wurtzite structure of ZnS. (**d**) shows the intergrowth of the two main polytypes of ZnS, zinc blende and wurtzite, within the same nanoparticle.

Further morphological analysis of the deposits was performed by TEM on samples directly deposited by PLD on TEM grids. Figure 4 shows representative images of the deposits at different magnification. As can be observed in Figure 4a, there is a large dispersion in the nanoparticle size, which ranges from few to tens of nanometers in diameter, in agreement with the AFM results shown above. High magnification TEM images (Figure 4b–d) of individual nanoparticles revealed the presence of the two polytypes of ZnS; zinc blende and wurtzite, in some cases co-existing in the same nanoparticle. In Figure 4b, recorded along the [0001]_W_ zone axis (see the FFT as inset) of a single nanoparticles, the wurtzite type structure with the characteristic hexagonal arrangement is observed. In Figure 4c a single nanoparticle of the zinc blende phase along the [001]_ZB_ zone axis is clearly identified through the FFT-fast Fourier transform (see inset in the upper right side of the panel) of the area of the nanoparticle squared in yellow. Finally, in Figure 4d an example of a nanoparticle where the intergrowth of the two polytypes of ZnS can be observed, being stacking faults on the ABCABC stacking of the zinc blende cubic phase the driving forces to yield a stacking of ABABAB layers of the hexagonal wurtzite structure. The yellow arrows point to areas corresponding to zinc blende oriented along [-110]_ZB_ zone axis (see the FFT inset in the upper left side of the image), whereas the green ones identify domains of wurtzite along the [2-1-10] zone axis (see the FFT inset in the upper right side of the image). The coexistence of the zincblende and wurtzite phases has been described before for nanostructured ZnS [24], where the role of surface energy becomes important and the phase transformation can happen at much lower transition temperatures than in the bulk. A closer analysis of the HRTEM image allows us to identify the presence of other defects in the form of dislocations mainly in the zinc blende domains. The presence of Co atoms was confirmed by transmission electron microscopy and energy dispersive X-ray microanalysis (TEM-EDX) analysis of individual crystalline nanoparticles (see table in Figure S2 of the Supplementary Materials).

**Figure 5:**
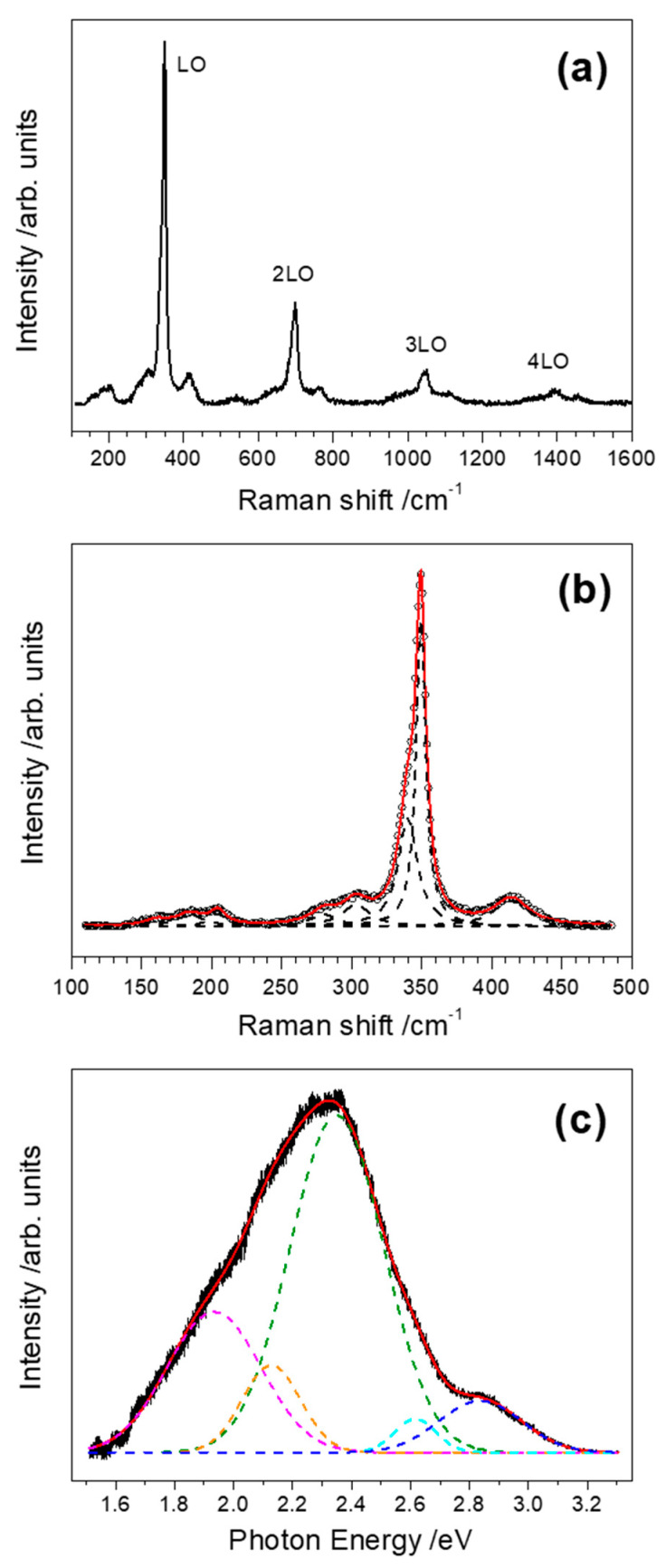
(**a**) Raman scattering spectrum of a Co:ZnS sample deposited on a Si substrate with 2 × 10^6^ double pulse sequences and an interpulse delay of 10 ps. (**b**) Lorentzian deconvolution of the Raman spectrum in the (100–500) cm^−1^ range. Circles represent the experimental data while the red solid line corresponds to the best-fit curve. (**c**) Room-temperature photoluminescence (PL) spectrum of the same sample. Gaussian deconvolution reveals emission bands centered at 2.84, 2.62, 2.35, 2.13, and 1.93 eV (dashed lines). The black line corresponds to the experimental data while the red solid line corresponds to the best-fit curve.

The Raman and photoluminescence (PL) response of the deposits upon excitation with a He-Cd laser at 325 nm was also evaluated. Near-resonant scattering is observed in the Raman spectrum plotted in Figure 5a, with a band at 349 cm^−1^ which is assigned to the longitudinal optical phonon (LO) of ZnS. Second, third and even fourth order of this mode are also observed (2LO, 3LO, and 4LO) [60,61], which indicates the formation of crystalline material, in agreement with HRTEM measurements. In addition, Lorentzian deconvolution of the spectrum in the (100–500) cm^−1^ range (Figure 5b) reveals the existence of weaker peaks centered at about 160, 185, 205, 280, 303, 340, and 414 cm^−1^. The peaks centered near 160 and 185 cm^−1^ can be assigned to disorder activated second order transversal acoustic (2TA) phonons [62,63], while the peak found near 205 cm^−1^ corresponds to the second order longitudinal acoustic (2LA) phonon mode in ZnS [64]. The peak observed at 280 cm^−1^ can be attributed to a transversal optical (TO) phonon [64,65]. The origin of the peak observed at 303 cm^−1^ has not been well established. According to Nilsen [66], it could be associated to an overtone originating from the W critical point on the zone boundary where the single phonon energies might deviate somewhat from those at the X and L critical points. The 340 cm^−1^ peak, which appears as a shoulder of the dominant LO peak, can be assigned to a surface optical (SO) phonon activated by a symmetry breaking mechanism associated with perturbation of the surface potential and has been previously observed in Raman spectra of ZnS nanoparticles, nanowires and nanobelts [65,67]. Finally, the 414 cm^−1^ peak is a second-order scattering process corresponding to a LO+TA phonon mode [65]. Raman peaks related to other compounds different from ZnS were not observed.

The PL spectrum of the Co:ZnS sample deposited on a Si substrate with 2 × 10^6^ double pulse sequences and an interpulse delay of 10 ps (Figure 5c) shows a broad emission between 1.6 and 3.2 eV that can be deconvoluted into five Gaussian peaks centered at 2.84 eV (437 nm), 2.62 eV (473 nm), 2.35 eV (528 nm), 2.13 eV (582 nm), and 1.93 eV (642 nm), respectively. These emissions are related to native defects and/or impurities which introduce different levels in the band gap [68]. Precisely, the 2.84 eV peak is commonly attributed to sulfur vacancies acting as donor states [69], while the 2.62 eV band corresponds to the so-called self-activated emission of ZnS. The origin of this luminescence is a donor-acceptor pair (DAP) transition. The acceptor is thought to be a complex called A-centre, which consists of a Zn vacancy and charge-compensating co-activator ion (frequently impurities or dopants such as Cl, Br, Al or Ga) at the next neighbor site, while the donor is shallow and located further away in the lattice [70,71]. Luminescence in the green range of the visible spectrum is a common feature in PL studies of either bulk or nanostructured ZnS samples. In the present case, a dominant 2.35 eV band is observed. Although there is a general agreement that such luminescence is due to a DAP transition, the origin of the defects involved has not been clearly ascertained. Zinc vacancies, sulfur interstitials and Cu impurities are frequently proposed as the acceptor levels, while possible donors include sulfur vacancies, Al, or Cl impurities [68,72,73,74]. The intensity of the green PL has been found to increase in Co-doped ZnS thin films with the dopant concentration, which is attributed to the role played by Co^2+^ ions as sensitizing agents enhancing the corresponding radiative recombination process [21]. The emission peak at 2.13 eV has been previously observed in both undoped and Mn-doped ZnS nanostructures but its origin remains unclear [69,75]. A band at exactly this energy has been also found in a recent cathodoluminescence study of ZnS nanobelts and correlated with an increased concentration of extended defects such as stacking faults [76] in agreement with our TEM measurements shown in Figure 4. Emission in the red range, like our 1.93 eV band, is usually attributed in ZnS to deep levels related to impurities such as Fe, Pb, Cu, In, or O [61,69,77].

**Figure 6:**
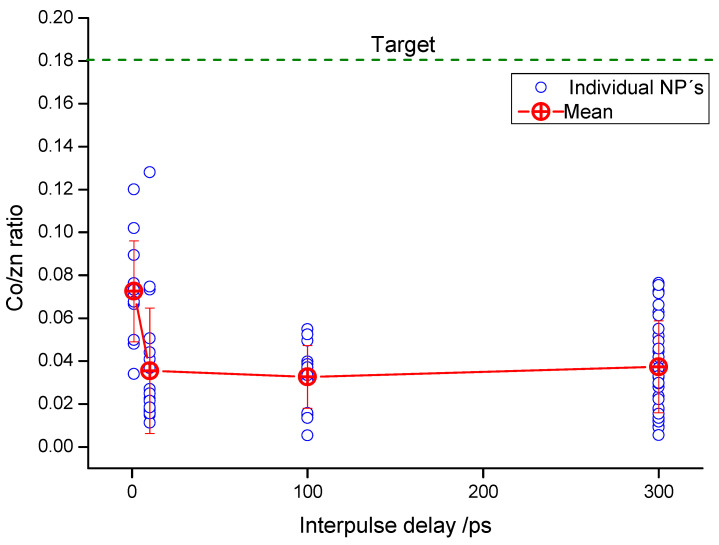
Cobalt to zinc ratios found in individual nanoparticles present in deposits prepared under DP irradiation as a function of the interpulse delay.

Finally, the possible effect of the interpulse delay on the composition of the deposits was assessed by analyzing the nanoparticles present in samples prepared by DP irradiation at different delays between the pump and the probe pulses. For that purpose, TEM coupled to EDX was used and the obtained Co/Zn ratio was chosen as a figure of merit to evaluate possible changes in the composition of the deposits with the delay. Figure 6 shows the Co/Zn atomic ratios obtained in individual nanoparticles found in different deposits as a function of the interpulse delay used for each film preparation. For the analysis, the electron beam waist was adjusted to the approximate diameter of the individual particle under study.

Besides the expected Co-doped particles with close to stoichiometric 1:1 Zn/S ratio, a small fraction of high Co content particles have been also found. This observation is particularly relevant in deposits obtained at the shortest interpulse delays. The proportion of nanoparticles found with substantial metallic character (Co proportion > 50%) is approximately 4% of the total. The appearance of metallic aggregates has been described in some previous works; in our case we believe that they may be due to local inhomogeneities in the distribution of compounds in the physically mixed target. Therefore, in order to avoid confusion, the graph in Figure 6 shows only the values of particles with Co/Zn ratios below the value of the original target (0.18) and those where the S/Zn ratio is within the range 1 ± 0.2. As is clear from Figure 6, the proportion of Co in the ZnS nanoparticles is significantly lower than the proportion of Co in the original mixed target. The obvious reason for this is that Co appears in part in the form of the metallic aggregates mentioned above. Significant dispersion is observable for the Co/Zn ratio for all PLD conditions employed. Interestingly, neither the average Co/Zn ratio nor its dispersion across nanoparticles seem to have a strong dependence on the delay between pulses in the double-pulse PLD experiment, except at 1 ps delay, where the average Co/Zn ratio is somewhat higher.

The results presented above demonstrate that complex irradiation schemes in the ultrashort temporal range allow for significant versatility in the pulsed laser deposition of doped semiconductor materials and offers intriguing avenues for further exploration, particularly in relation with the role of the detailed characteristics of the original target on the inclusion of the dopant in the host matrix, or with the exploration of a broader class of temporal pulse shapes beyond double pulse irradiation.

## 4. Conclusions

Co-doped ZnS crystalline nanoparticles have been obtained by double pulse femtosecond ablation of a mixture of ZnS and Co (10%) and deposition on Si substrates and TEM grids at room temperature under vacuum conditions. The analysis of the deposits morphology by reflectance measurements and AFM provides evidence on the effect of the double pulse irradiation scheme. These results indicate that while the individual pulses are kept close to the ablation threshold, the combined effect of two consecutive pulses produced a measurable deposit even at the longer interpulse delay studied (300 ps). This is indicative of a coupling effect on the material as observed in previous studies of the ablation plasma by TOF-MS in a similar DP configuration. This coupling effect is stronger at shorter interpulse delays; hence a thicker deposit is observed for samples prepared by DP irradiation with an interpulse delay of 10 ps. These results are corroborated by the thickness analysis performed by AFM. The composition and crystallinity of the nanoparticles forming the deposits was studied by TEM and TEM coupled to EDX analysis. These measurements revealed the crystalline nature of the nanoparticles, finding structures corresponding to the two polytypes of ZnS, wurtzite and zinc blende. The compositional analysis confirmed the presence of Co in individual crystalline nanoparticles. Inspection of the deposits by Raman spectroscopy indicates the formation of a crystalline material, in agreement with TEM results. Raman peaks related to compounds different from ZnS are not detected. Regarding the PL response, luminescence is observed in the green and in the red range and these emissions are attributed to native defects and/or impurities. Finally, the possible influence of the interpulse delay on the deposits composition was assessed by comparing the composition of nanoparticles found in deposits prepared at different interpulse delays. These results show that the average Co/Zn ratio is lower than that expected from purely stoichiometric transfer from the physically mixed target. Both this ratio and its dispersion across different nanoparticles are stable with respect to the delay between pulses.

## Figures and Tables

**Table 1:** Averaged thickness values of the deposits obtained by DP irradiation with a train of 10^6^ pairs of pulses at different interpulse delays (Δt) and by SP irradiation with a train of 10^6^ pulses.

Δt (ps)	Thickness (nm)
10	66 ± 8
100	57 ± 7
300	43 ± 9
SP	8 ± 2

## Supplementary Materials

The following are available online at https://www.mdpi.com/2079-4991/10/11/2229/s1, Figure S1: Variation of the reflectance as a function of the deposit thickness, Figure S2: TEM image of an individual crystalline nanoparticle analysed by EDX.
