# Supplementary material for: Femtosecond Double-Pulse Laser Ablation and Deposition of Co-Doped ZnS Thin Films
Source: Nanomaterials (Basel). 2020 Nov 10;10(11):2229. doi: 10.3390/nano10112229 (PMC7698319; doi:10.3390/nano10112229)
Supplement: Supplementary file 1 [file nanomaterials-10-02229-s001.pdf]

Supplementary information

Femtosecond Double-Pulse Laser Ablation and Deposition of Co-Doped ZnS Thin Films

Ignacio Lopez-Quintas [1,†,*], Esther Rebollar [1], David Ávila-Brande [2], Jesús G. Izquierdo [3], Luis Bañares [3], Carlos Díaz-Guerra [4], Ana Urbieta [4], Marta Castillejo [1], Rebeca de Nalda [1,*] and Margarita Martín [1]

[1] Instituto de Química Física "Rocasolano", Agencia Estatal CSIC, Serrano 119, 28006 Madrid, Spain; e.rebollar@csic.es (E.R.); marta.castillejo@iqfr.csic.es (M.C.); mmm@iqfr.csic.es (M.M.)

[2] Departamento de Química Inorgánica, Universidad Complutense de Madrid, 28040, Madrid, Spain; davilabr@ucm.es

[3] Departamento de Química Física, Universidad Complutense de Madrid, 28040, Madrid, Spain; jegonzalez@ucm.es (J.G.I.); lbanares@ucm.es (L.B.)

[4] Departamento de Física de Materiales, Facultad de Ciencias Físicas, Universidad Complutense de Madrid, Ciudad Universitaria s/n, 28040, Madrid, Spain; cdiazgue@ucm.es (C.D.-G.); anaur@ucm.es (A.U.)

* Correspondence: ilopezquintas@usal.es (I.L.-Q.); r.nalda@csic.es (R.d.N.)

† Present address: Grupo de Investigación en Aplicaciones del Láser y Fotónica, Departamento de Física Aplicada, University of Salamanca, 37008 Salamanca, Spain

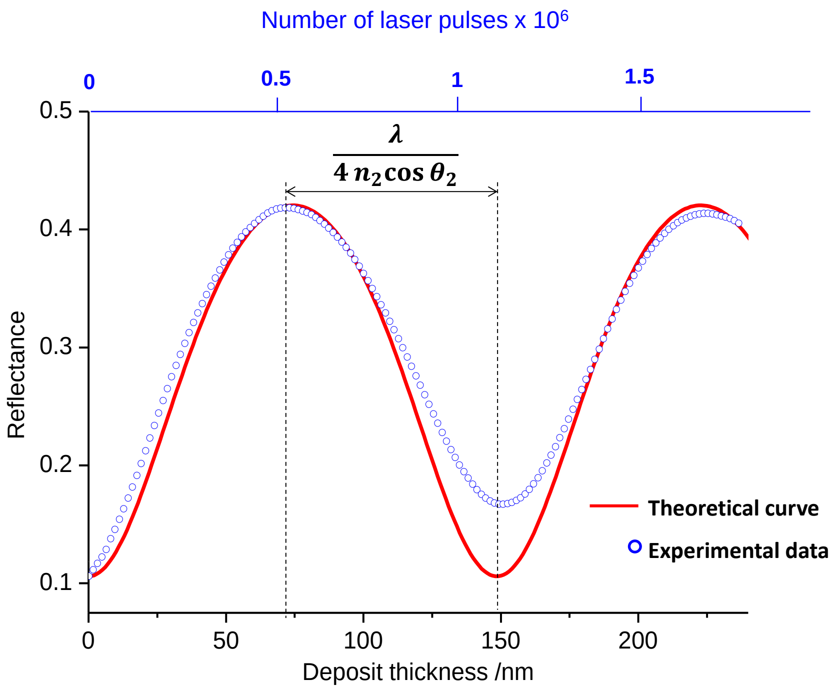

Figure S1. Variation of the reflectance as a function of the deposit thickness. The red line represents the theoretical reflectance curve expected for an incidence angle θ_1 = 82.7°. The blue circles represent the experimental reflectance data obtained during the deposition of Co/ZnS on a silicon substrate by DP ablation at Δt = 2 ps using a fluence of 0.8 J cm^{-2} for each individual pulse.

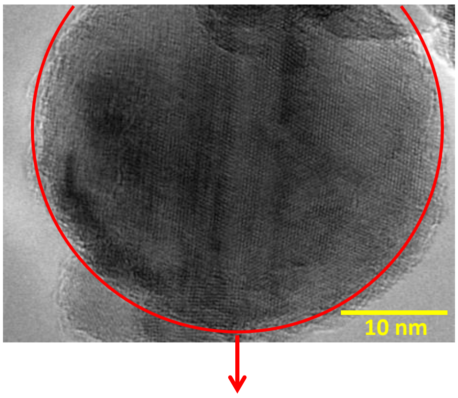

	S	Co	Zn
Atomic %	45.7	1.6	52.8

Figure S2. TEM image of an individual crystalline nanoparticle analysed by EDX. The red circle delimitates the approximate area analyzed. In the table, the corresponding atomic percentage of S, Co and Zn found in the nanoparticle is given. The deposit was obtained by PLD with DP irradiation at an interpulse delay of 300 ps and $2x10^6$ pulses.
